# Pleural mesothelioma in a patient with Familial Mediterranean Fever: A case report

**DOI:** 10.5339/qmj.2026.20

**Published:** 2026-03-31

**Authors:** Asma Albtoosh, Omar Wael Alhasan, Mohammad Alakhras, Luma Taweel, Baraa Ayed Al Odat, Maram Abdaljaleel, Husam Abuawad, Oday Abu Ajamieh, Tareq Gharaibeh, Khalid Oweidat, Samer Al Sawalhi

**Affiliations:** 1 Department of Respiratory and Sleep Medicine, School of Medicine, The University of Jordan, Amman, Jordan; 2 School of Medicine, The University of Jordan, Amman, Jordan; 3 Department of Internal Medicine, School of Medicine, The University of Jordan, Amman, Jordan; 4 Department of Pathology, Microbiology and Forensic Medicine, School of Medicine, The University of Jordan, Amman, Jordan; 5 Department of Internal Medicine, Specialty Hospital, Amman, Jordan; 6 Thoracic Surgery Department, Specialty Hospital, Amman, Jordan *Email: skyscraper555@yahoo.com

**Keywords:** Mesothelioma, pleural mesothelioma, Familial Mediterranean Fever, pleural effusion, Jordan, case report

## Abstract

**Background:**

Malignant mesothelioma is a rare, lethal neoplasm of mesothelial surfaces, classically attributed to asbestos exposure. However, as regulations have reduced asbestos use, there is an increased focus on non-asbestos-related causes. Among these, the chronic serosal inflammation characteristic of Familial Mediterranean Fever (FMF) has been reported in a few cases. Although a direct causal relationship has not yet been established, such cases are critical for identifying the potential long-term risks of chronic serosal inflammation. Here, we present the fourth documented case of pleural mesothelioma associated with FMF.

**Case presentation:**

A 55-year-old male patient with known FMF was admitted to the hospital with progressive shortness of breath and cough for one month. Physical examination and chest X-ray revealed a large left-sided pleural effusion. Thoracocentesis analysis demonstrated an exudative effusion with predominant lymphocytosis. A contrast-enhanced computed tomography (CT) scan showed pleural thickening with enlarged mediastinal lymph nodes. Positron emission tomography (PET) scan revealed avid pleural uptake (SUVmax = 7.5). A pleural biopsy was therefore performed, revealing epithelioid pleural mesothelioma. The patient had no known history of asbestos exposure. However, he was poorly compliant with colchicine treatment and experienced frequent episodes of serositis, occurring on average twice monthly. The patient underwent surgical resection followed by chemotherapy and is doing well 10 months after the initial presentation.

**Discussion:**

Many cases of FMF have been reported in association with peritoneal mesothelioma, but few have been linked to pleural mesothelioma. In this case, we highlight that chronic serosal inflammation, characteristic of untreated FMF, may represent a potential risk factor for non-asbestos-related malignant pleural mesothelioma. Larger-scale registry studies may be required to establish a statistically significant association.

**Conclusion:**

This case reinforces the hypothesis that uncontrolled FMF may predispose patients to malignant mesothelioma. The presence of such an association would further stress the importance of early recognition and management of FMF.

## 1. INTRODUCTION

Mesothelioma is a rare malignant neoplasm that arises from mesothelial cells lining the serosal cavities. Its incidence is 0.43 per 100,000 people, with approximately 80% of cases involving the pleura.^1,2^ It generally occurs beyond the seventh decade and carries a poor prognosis, with an overall survival of approximately 10.3 months.^3^ Although asbestos exposure is the most significant risk factor, other etiologies, such as fibrous silicates and genetic factors, have also been documented.^4^

Familial Mediterranean Fever (FMF) is an autosomal recessive disorder characterized by recurrent episodes of fever and serositis, including peritonitis, pleuritis, pericarditis, synovitis, and erysipelas-like erythema.^5^ While colchicine is the gold standard for preventing these acute inflammatory episodes and mitigating amyloidosis, its therapeutic efficacy depends on consistent administration.^6^ Interruption of colchicine therapy is linked to a higher frequency of FMF attacks, which subjects the mesothelium to chronic inflammatory stress. This environment leads to elevated levels of pro-inflammatory cytokines, including interleukin (IL)-1β, IL-6, and tumor necrosis factor-alpha (TNF-α), which are potentially carcinogenic and may play a role in driving malignant mesothelial transformation.^7^

The patient was managed in Jordan University Hospital, a tertiary academic center in Amman, Jordan. Institutional Review Board (IRB) approval was obtained for this case report, and informed consent was obtained from the patient for the publication of his case details and associated images.

## 2. CASE PRESENTATION

A 55-year-old man, diagnosed with FMF, self-presented to the emergency department with progressive shortness of breath and dry cough over the past month. He denied fever, sweating, or chest pain but reported loss of appetite and perceived weight loss over the preceding couple of months. His FMF diagnosis was made clinically in childhood, with genetic testing later confirming a homozygous M694V mutation in the Mediterranean Fever (MEFV) gene. Colchicine was prescribed at a dose of 1 mg once daily; however, he was poorly adherent and experienced frequent episodes of serositis—manifesting as chest and abdominal pain—occurring 2–3 times per month. He is an ex-smoker, having smoked one pack daily for 16 years, and quit 25 years ago. He is a retired land surveyor with no history of asbestos or silica exposure.

The patient’s oxygen saturation was 93% on room air, with a respiratory rate of 22 breaths per minute. His heart rate was 95 beats per minute, blood pressure was 132/72 mmHg, and temperature was 36.7°C. Lung auscultation revealed decreased air entry over the entire left lung field. A chest X-ray revealed a massive left-sided pleural effusion. A 10 French pigtail catheter was inserted, and 450 mL of serous pleural fluid was drained initially. Pleural fluid analysis showed an exudative lymphocytic effusion with the following results: white blood cells count of 332 cells/mm³ (93% lymphocytes), pleural protein 5.6 g/dL, serum protein 7.4 g/dL (protein ratio 0.75), pleural lactate dehydrogenase (LDH) 125 U/L, and serum LDH 234 U/L (LDH ratio 0.53). Cytological examination of five separate pleural fluid samples was negative for malignancy. Gram stain, cultures, Ziehl–Neelsen stain, acid-fast bacilli smear, and mycobacterial culture were all negative.

Based on the high-output pleural drainage of approximately 500 mL daily during the first three days, a pan-CT scan was performed to rule out any underlying malignant process, specifically lymphoma. The scan revealed left pleural thickening, residual pleural effusion, and enlarged mediastinal lymph nodes, with the largest measuring 1 cm in the paratracheal region. The rest of the scan was unremarkable. Given the patient’s long-standing and severe FMF history, an abdominal fat pad biopsy was also performed the following day and was negative for amyloidosis.

A positron emission tomography (PET) scan was performed two days later. The scan revealed hypermetabolic nodular thickening of the left lower pleura extending to the pleuroperitoneal reflection (SUVmax = 7.5), along with an ipsilateral pleural effusion. Mild hypermetabolic activity was observed in the left internal mammary lymph node (SUVmax = 2.9), and several thoracic and cervical lymph nodes showed minimal, non-specific metabolic activity.

**Figure 1.**
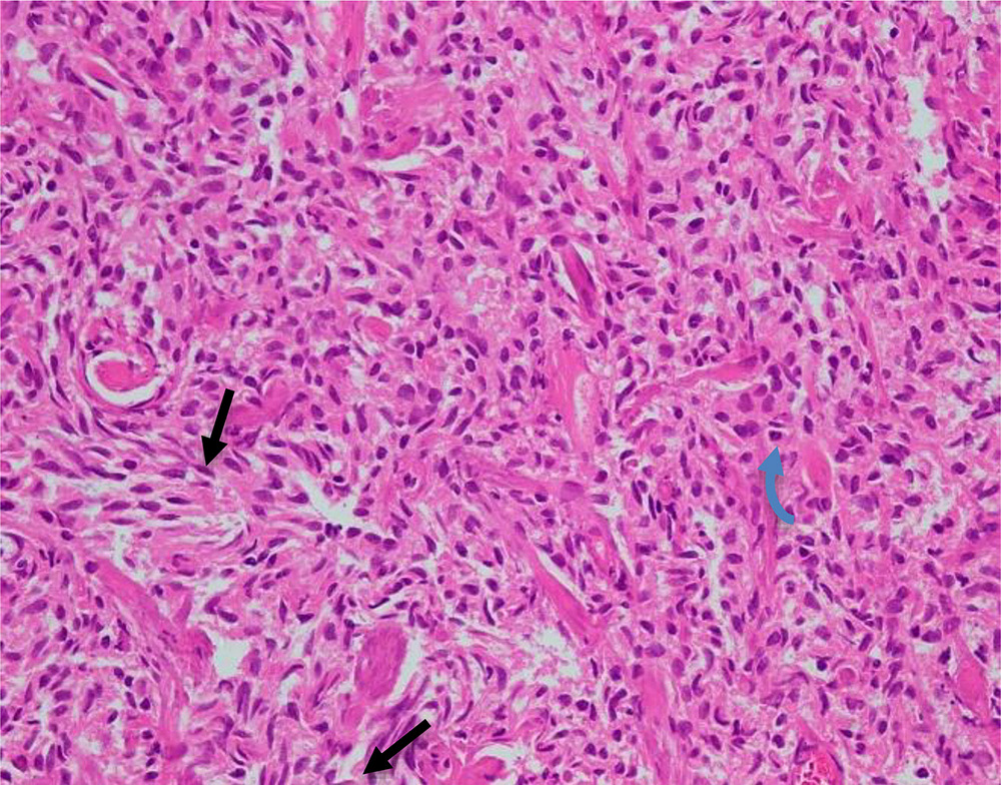
Histopathology of the pleural biopsy (H&E stain, 400×). The figure reveals spindle (straight black arrows) and round (curved blue arrows) morphologies of malignant cells infiltrating the fibrovascular stroma. The cells are characterized by hyperchromatic nuclei and irregular nuclear contours. These histological features support the diagnosis of malignant pleural mesothelioma.

**Figure 2.**
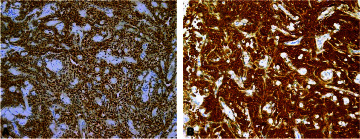
Immunohistochemical staining. (A) Podoplanin (D2-40) and (B) calretinin show diffuse positive expression (brown staining) in the infiltrating cells, with small areas of negative intervening fibrous and vascular structures (blue staining). The combination of positive calretinin and podoplanin expression confirms the mesothelial origin of the tumor cells.

The patient was scheduled for video-assisted thoracoscopic surgery (VATS) for a pleural biopsy three days later, and histopathology confirmed a diagnosis of malignant pleural mesothelioma (MPM) of the epithelioid type (Figure 1). Immunohistochemistry was positive for calretinin, D2-40, and WT1, and negative for epithelial membrane antigen (EMA), desmin, GLUT-1, and p53 (Figure 2).

The patient was referred to a specialized cancer center for management. He underwent an extended pleurectomy and left hemidiaphragm resection with reconstruction, followed by four cycles of carboplatin and pemetrexed. A CT scan performed one month after chemotherapy showed regression of the cervical and mediastinal lymph nodes. The patient was subsequently started on radiotherapy and was last seen doing well 10 months after the initial presentation.

## 3. DISCUSSION

FMF is an autosomal recessive autoinflammatory disorder characterized by recurrent episodes of serositis, primarily affecting the peritoneum, pleura, and synovium. It is caused by mutations in the MEFV gene, located on the short arm of chromosome 16. These mutations lead to a gain-of-function in the pyrin protein, resulting in inflammasome dysregulation and propagation of the inflammatory cascade, which triggers the secretion of various cytokines.^8^

MPM is a rare and aggressive tumor typically associated with asbestos exposure, though its prognosis remains poor due to delayed diagnosis and limited treatment options.^9^ Chronic serosal inflammatory conditions, such as FMF, have been discussed as non-asbestos causes of MPM, although FMF has not yet been definitively established as a risk factor for mesothelioma.^4^ While the overall cancer risk in FMF patients is reduced, the low incidence of mesothelioma (0.13–2.33 per 100,000) in the general population likely limits its detection in studies with fewer than 10,000 patients.^9,10^

Chronic inflammation, as seen in FMF, is a well-established driver of malignancy. Inflammatory cytokines such as IL-1β and IL-6, which are elevated during FMF attacks, have been implicated in cancer development, with emerging evidence linking chronic inflammation to carcinogenesis.^6^ Additionally, TNF-α, a key cytokine in FMF pathogenesis, is thought to play a significant role in the carcinogenic effects of asbestos on mesothelial cells.^11^ In the present case, the patient’s homozygous M694V genotype, along with his noncompliance to colchicine, resulted in more frequent inflammatory attacks and a prolonged inflammatory state, which, combined with the absence of asbestos exposure, may have contributed to the development of MPM.

Pleural effusion, a known complication of FMF, may delay the diagnosis of mesothelioma if not carefully considered and excluded. In the present case, five pleural fluid cytology samples were negative for malignancy, consistent with the low sensitivity of cytology for mesothelioma diagnosis.^12^ This underscores the diagnostic challenge, as an accurate diagnosis of mesothelioma often requires more invasive procedures, such as VATS, which has a sensitivity of 95%.^13^

While many cases of mesothelioma in patients with FMF have been reported in the literature, only three involved pleural mesothelioma.^14–16^ The prolonged state of inflammation present in this case was also observed in the other reported cases: Challita et al. and Kon et al.’s patients were colchicine-resistant, predisposing them to frequent FMF attacks preceding the development of the epithelioid subtype of MPM, which was also seen in our case.^14,15^ Their prognosis was poor, as two of the three cases died within a year of presentation, with the third alive at five months after presentation when reported. These cases suggest a relationship between the chronic inflammatory state seen in FMF and the development of mesothelioma. In our case report, we present the fourth reported case of MPM in a patient with FMF and explore the possible association between the two.

## 4. CONCLUSION

Pleural mesothelioma is a rare malignant neoplasm with a poor prognosis, traditionally linked to asbestos exposure. However, cases without significant asbestos exposure have been associated with chronic inflammatory states, such as FMF. Persistent inflammation in untreated FMF patients may contribute to the development of mesothelioma through cytokine-mediated mechanisms. Clinicians should remain vigilant regarding the importance of colchicine adherence in patients with FMF to suppress the chronic inflammatory state and potentially mitigate long-term risks. In the present case, early surgical resection followed by systemic chemotherapy resulted in favorable short-term clinical outcomes, with the patient remaining well 10 months after diagnosis.

## COMPETING INTERESTS

The authors have no conflicts of interest to declare.

## AUTHOR CONTRIBUTIONS

All authors contributed to the conception and design of the study and drafted the manuscript, including giving final approval to the manuscript version submitted for publication. All authors read and approved the final manuscript.
